# Perceptual judgment and saccadic behavior in a spatial distortion with briefly presented stimuli.

**DOI:** 10.2478/v10053-008-0072-6

**Published:** 2010-02-11

**Authors:** Sonja Stork, Jochen Müsseler, A. H. C. van der Heijden

**Affiliations:** 1 Department of Psychology, Ludwig Maximilian University Munich, Germany; 2 Department of Psychology, RWTH Aachen University, Germany; 3 Faculteit der Sociale Wetenschappen, Instituut Psychologie, Leiden University, The Netherlands

**Keywords:** eye movement, saccade, localization, position, absolute position judgement, relative position judgement, space perception, visual illusion

## Abstract

When observers are asked to localize the peripheral position of a small probe with respect to the mid-position of a spatially extended comparison stimulus, they tend to judge the probe as being more peripheral than the mid-position of the comparison stimulus. This relative mislocalization seems to emerge from differences in absolute localization, that is the comparison stimulus is localized more towards the fovea than the probe. The present study compared saccadic behaviour and relative localization judgements in three experiments and determined the quantitative relationship between both measures. The results showed corresponding effects in localization errors and saccadic behaviour. Moreover, it was possible to estimate the amount of the relative mislocalization by means of the saccadic amplitude.

## INTRODUCTION

Spatial acuity is known to be of high precision when measured under optimal viewing conditions with a temporally extended stationary stimulus with high contrast (for overviews, see e.g., Skavenski, 1990; Westheimer, 1981). Spatial acuity is much poorer when measured with a stimulus of short duration and low contrast (see e.g., Bedell & Flom, 1983; Bocianski, Müsseler, & Erlhagen, 2008; Leibowitz, Myers, & Grant, 1955; Mateeff & Gourevich, 1983; Mateeff & Hohnsbein, 1988; O’Regan, 1984; Rose & Halpern, 1992). Moreover, localization is distorted when stimuli are briefly presented before, during, or after a saccade or during smooth pursuit eye movements (e.g., Awater & Lappe, 2006; Brenner, Smeets, & van der Berg, 2001; Rotman, Brenner, & Smeets, 2005).

Müsseler and colleagues (Müsseler & van der Heijden, 2004; Müsseler, van der Heijden, Mahmud, Deubel, & Ertsey, 1999; van der Heijden, Müsseler, & Bridgeman, 1999) also investigated spatial localization under less than optimal viewing conditions. The observers were asked to judge the peripheral position of a small probe with respect to the mid-position of a spatially extended comparison stimulus. When the two stimuli were flashed successively a systematic deviation was consistently observed: The observers perceived the probe as being more peripheral than the mid-position of the comparison stimulus.

**Figure 1.**
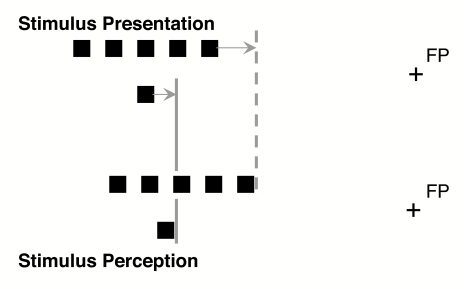
Stimulus presentation and stimulus perception in the relative localization task. The greater outer localization of the single lower square (the probe) relative to the mid-position of the spatially extended row of the upper squares (the comparison stimulus) is assumed to emerge from two different foveal tendencies of the comparison stimulus (shifted to the dashed line) and the probe (shifted to the straight line). FP = fixation point.

To explain this relative mislocalization, Müsseler and colleagues (Müsseler & van der Heijden, 2004; Müsseler et al., 1999) assumed it emerged from different absolute localizations of the probe and mid-location of the comparison stimulus. From the literature it is already well-known that the absolute location of a briefly presented target is often perceived more foveally than it actually is (see e.g., Kerzel, 2002; Mateeff & Gourevich, 1983; Müsseler et al., 1999, Experiment 4; O’Regan, 1984; Osaka, 1977; van der Heijden, van der Geest, de Leeuw, Krikke, & Müsseler, 1999). In order to explain the relative mislocalization we assumed that a spatially extended stimulus is localized even more foveally than a spatially less-extended probe. Then the probe’s relative position is perceived as more peripheral than the mid-position of the comparison stimulus (see Figure 1). This explanation of the relative mislocalization was successfully tested against alternative accounts (for details, see Müsseler & van der Heijden, 2004; Müsseler et al., 1999).

The assumptions made by Müsseler and colleagues, and especially the assumption that a spatially extended stimulus is localized more foveally than a spatially less extended probe, certainly need some supporting evidence. In this context it is of importance to know that comparable foveal tendencies in absolute localizations are found in saccadic eye movement studies. Firstly, saccades tend to undershoot a peripheral target by about 5–10% of its eccentricity – an error that is normally compensated with a corrective saccade (see e.g., Aitsebaomo & Bedell, 1992; Bischof & Kramer, 1968; Lemij & Collewijn, 1989). Secondly, the saccadic undershoot seems to increase with spatially extended stimuli (so-called *centre-of-gravity effect*; cf. Findlay, Brogan, & Wenban-Smith, 1993; see also Vos, Bocheva, Yamimoff, & Helsper, 1993). Moreover, the size of the saccadic undershoot is in the same range as the size of the foveal mislocalization observed in a perceptual judgement task (see van der Heijden, van der Geest, et al., 1999). So, saccadic eye movement research provides support for assumptions of Müsseler et al. (1999).

The comparability between eye-movement behaviour and perceptual judgement tasks suggests an intriguing possibility: The possibility that the saccadic eye movement system is at the basis of, and provides the information for, position judgements in position-judgement tasks (see also e.g., van der Heijden, Müsseler, & Bridgeman, 1999; Wolff, 1987, for this suggestion). With regard to this possibility it is of importance to know that, in addition to the pattern of undershoot that saccades and localization judgements apparently have in common, there are further correspondences between saccadic eye movements and localization judgements. Four points are worth mentioning here.

The first point concerns the effect of exposure duration. It is well established that both saccadic eye movements and localization judgements become more precise with longer exposure durations of a target (e.g., Abrams, Meyer, & Kornblum, 1989; Aitsebaomo & Bedell, 1992; Kowler & Blaser, 1995; Lemij & Collewijn, 1989).

The second point concerns the effect of grouping within the stimulus array. It is well-known that the amplitude of saccades to targets depends on the grouping within a stimulus array; if one element is made larger (Findlay, 1982), is made more intense (Deubel, Wolf, & Hauske, 1984), or is presented with higher contrast (Deubel & Hauske, 1988), the saccade lands closer to that target. The results obtained with a relative localization experiment are in line with these findings. A salient square placed at either the inner or the outer edge of a comparison stimulus affects relative mislocalization as it affects saccadic behaviour; with the salient square at the outer position the probe is perceived as more peripheral than with the salient square at the inner position (see Müsseler et al., 1999, Experiment 7).

Third, recent studies demonstrated an effect of saccadic adaptation on pointing and verbal localization, that is a shift in the direction of adaptation (Bruno & Morrone, 2007; Collins, Doré-Mazars, & Lappe, 2007; Georg & Lappe, 2009). On the basis of these results the authors suggested that a common mechanism might serve to recalibrate both the perceptual and the action map and that the system providing saccade metrics also contributes to the metric used for space perception.

The last – but probably not least – point concerns the effect of stimulus onset asynchrony (SOA) between comparison stimulus and probe in a relative judgement task. The relative mislocalization emerges in an interval in which saccadic eye movements are programmed and executed, that is typically between 50 and 200 ms (Müsseler et al., 1999, Experiment 2).

Taken all together, the similarities between saccadic eye-movement behaviour and localization judgements are quite suggestive. So, there is evidence that the saccadic eye movement system is at the basis of and provides the information for the localization judgements. Nevertheless, there are at least three reasons to be careful about accepting this assumption.

Firstly, eye movements were not measured directly in the relative judgement tasks under discussion. The evidence for a close correspondence between saccadic eye movement behaviour and position judgements comes from different studies designed for different purposes.

Secondly, although the correspondence seems to be obvious at first sight, other observations cast doubt on a too strong relationship between saccadic eye movements and spatial localization judgements. Recently several spatial dissociations between motor behaviour (including eye movements) and perception have been reported and are still under discussion (for an overview, see Rossetti & Pisella, 2002).

Thirdly, different brain areas with different spatial maps are involved in perception and in the programming of saccadic eye movements. Visual information can reach the brainstem oculomotor centres by several routes: directly from the retina via the superior colliculus; from a route via the corpus geniculatum laterale, the primary striate cortex, and the superior colliculus; from a route via the corpus geniculatum laterale, the visual cortex, and the frontal eye fields; and last – but probably not least – from a route via the corpus geniculatum laterale, striate, prestriate and parietal cortices, and the frontal eye fields (cf. Deubel, 1999, p. 716). This multiplicity means that it is far from clear whether the spatial map used in perceptual judgement tasks corresponds metrically with the spatial map(s) involved in the programming of saccadic eye movements.

In fact, there are also studies showing a non-correspondence between a (saccadic) pointing task and a relative judgement task (e.g., Eggert, Sailer, Ditterich, & Straube, 2002; Müsseler, Stork, & Kerzel, 2008). For example, Eggert and co-workers examined the effect of distractor presentation on the relative spatial judgement and on the width of the primary saccadic amplitude. They found no correspondence between both measures. However, their general procedure differed from the spatial illusion, on which we focus here. Therefore, the aim of the present study was to examine whether saccading to the mid-position of the spatially extended comparison stimulus and saccading to the probe revealed more absolute foveal mislocalizations for the comparison stimulus than for the probe. Moreover, our aim is to compare quantitatively the amplitude of the saccadic behaviour with the location error of the relative judgement task.

Consequently, in three experiments two tasks are compared: In the relative judgement tasks, participants were asked to judge the perceived position of a probe relative to the mid-position of a comparison stimulus. This task matches the procedure used by Müsseler and colleagues (1999; see also Müsseler & van der Heijden, 2004). In the saccade task, participants were asked to execute a saccade to the probe or the mid-position of the comparison stimulus. In Experiment 1, relative judgements and saccadic amplitudes to the stimuli were compared. Experiments 2 and 3 were run in order to check whether different effects of eccentricity could be observed with both tasks.

## EXPERIMENT 1

Empirical evidence and theoretical considerations allow us to suggest that the relative mislocalization under consideration originated from localizing a spatially extended stimulus more towards the fovea than a spatially less-extended probe. This assumption was already successfully examined by an experiment with absolute mouse pointing, in which both stimuli were presented blockwise as single targets (Müsseler et al., 1999, Experiment 4). Additionally, if our assumption is correct that saccadic eye movements are at the basis of the mislocalization, we expect corresponding results in a saccadic eye-movement task. Therefore, Experiment 1 aims to compare the findings of the relative judgement task with the findings on saccadic behaviour in similar experimental situations.

The relative judgement task was basically identical to the procedure introduced by Müsseler et al. (1999). The probe and comparison stimulus were presented with an SOA of 0 and 120 ms. When both stimuli are flashed simultaneously, they can be processed in one spatial map as a single stimulus configuration. Therefore, with simultaneous presentation the position judgement of the probe relative to the comparison stimulus is expected to be more or less error-free. When the two stimuli are separated by an SOA, however, two successive configurations with different spatial information have to be superimposed. Then relative mislocalizations are expected to emerge (see Müsseler et al., 1999; Müsseler & van der Heijden, 2004).

The saccadic eye-movement task was basically identical to the procedure used in single-stimulus studies in basic saccadic eye-movement research. The comparison stimulus and probe were presented as single stimuli in a blocked sequence. If the relative judgement task and the saccade task correspond, a more pronounced eye-movement undershoot to the spatially extended comparison stimulus than to the less extended probe is expected. Eye-movement studies already indicated comparable tendencies, that is larger undershoots with a spatially extended stimulus than with a less extended stimulus (see e.g., Findlay et al., 1993). The relevant experiments were, however, designed for different purposes and used in different experimental situations.

### Method

#### Apparatus and stimuli

The experiment was carried out in a dimly lit room. The experiment was controlled by a Macintosh computer and the stimuli were presented on a 17” colour monitor with black-on-white projection (832 x 624 pixels). The monitor had a refresh rate of 75 Hz and a luminance of approximately 40 cd/m². The participant’s head was placed on a chin and forehead rest 500 mm in front of the monitor.

**Figure 2.**
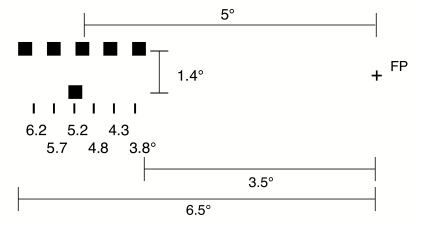
Stimulus presentation in the experiments. Participants fixated a cross in the middle of the screen. A single lower square (probe) and a spatially extended row of upper squares (comparison stimulus) appeared to the left or to the right of the fixation cross (here, 5° to the left). Participants were asked to judge the probe position (presented at 3.8°–6.2°) relative to the comparison stimulus's mid-position. FP = fixation point.

The stimuli appeared either to the left or to the right of a fixation cross. A square of 0.33° x 0.33° visual angle was used as the probe. A spatially more extended stimulus of 3° consisting of five squares, each separated from the next by 0.33°, was used as the comparison stimulus (see Figure 2). Stimuli were presented for only one frame of the monitor (13 ms).

In the relative judgement task, the comparison stimulus appeared 1.4° above the probe and its position was held constant at 5° (mid-position of the central square). The position of the probe was varied with respect to the mid-position of the comparison stimulus by ± 0.2°, ±0.7°, and ±1.2°; thus, it was presented at 3.8°, 4.3°, 4.8°, 5.2°, 5.7°, and 6.2° eccentricity.

In the saccade task either the comparison stimulus or the probe was presented. These stimuli appeared horizontally in line with the fixation cross. The stimuli were presented at the same positions as in the relative judgement task, that is between 3.8° and 6.2° eccentricity.

#### Design

The relative judgement task and saccadic eye-movement task were presented in separate blocks. The sequence of the blocks was counterbalanced over participants.

In the judgement task, the probe and comparison stimulus were presented in either the left or the right hemifield. They either appeared simultaneously or the comparison stimulus preceded the probe stimulus by an SOA of 120 ms. All combinations of hemifield (left, right), probe position (3.8 to 6.2°), and SOA (0, 120 ms) were presented in a randomized sequence. In total, participants were confronted with 192 trials in the judgement task.

In the saccade task, the comparison stimulus and the probe were presented blockwise in a counterbalanced order. Again, all participants were confronted with 192 presentations of the stimuli in the left and right hemifields.

#### Procedure

In the judgement task, participants initiated the stimulus presentation by simultaneously pressing the upper and lower key of a horizontally arranged computer mouse. Each trial began with an auditory signal and a central fixation cross that appeared for 1 s. The stimuli were presented for one frame (13 ms) 200 ms after the fixation point had vanished (this interval was introduced in order to facilitate the generation of eye movements in the saccade task, cf. Kingstone & Klein, 1993).

The instruction for the judgement task stressed that the participant should fixate the fixation cross when it appeared and not move the eyes after the cross had vanished. As the presentation of comparison stimulus and target was much too short to execute eye movements successfully and as keeping fixation was much more convenient for the observers than moving their eyes, eye movements were not recorded in the judgement task.^1^ After the presentation of the stimuli the observers had to answer the question “Which stimulus was more peripheral? The upper or lower?” by pressing the upper or lower mouse key. Following the key-press, the next trial was initiated with a programmed one-second delay. Participants received no feedback concerning their performance. To familiarize participants with the task, proper training trials were presented before the experiment.

In the saccade task, conditions were identical to the judgement task except that either only the probe or only the comparison stimulus was presented in the left or right hemifield. The participants were instructed to execute a saccade to the target as fast as possible, that is, to the probe or to the mid-position of the comparison stimulus, and to maintain fixation until the fixation cross reappeared. Then observers initiated the next trial via a button press. The experiment lasted approximately 90 min, including calibrations, training trials, and short breaks.

#### Measurement of eye movements

The horizontal position of the left or right eye was monitored with a head mounted infrared light reflecting eye-tracking device (Skalar Medical B.V., IRIS Model 6500). The eye movement modulated signal was band-pass, demodulated, and low-pass filtered (DC -100 Hz, -3dB) and then digitized at a rate of 250 Hz with a second Macintosh computer. By analysing the eye-movement signal, the saccadic onset was determined as the point in time where the ocular velocity exceeded 37.5°/s.

Calibration of the horizontal eye movements was accomplished by having the participant fixate at five evenly spaced dots across the screen. Calibrations were obtained by computing the linear regression for the five target locations. The computed gain was used in order to compute the saccadic amplitude. The calibration was repeated after every block (24 trials) of the experiments.

#### Participants

Sixteen female and 9 male individuals who ranged in age from 18 to 37 years (mean age of 24.4 years) were paid to participate in the experiment. All participants in the present and subsequent experiments reported normal or corrected-to-normal vision and were naive as to the purpose of the experiment.

### Results

As the dependent variable in the judgement task, the point of subjective equality (PSE, 50% threshold) between the probe and the mid-position of the comparison stimulus was computed by a probit analysis for every participant and condition (cf. Finney, 1971; Lieberman, 1983). As dependent variable in the saccade task the mean deviation between the eye’s first landing position and the real target position was calculated for every participant and condition. Three participants were excluded because their mean PSE values or saccadic amplitudes deviated more than ±2 standard deviations from the corresponding means of the sample. The mean saccade latency was 227 ms (*SE* = 12) for the comparison stimulus and 226 ms (*SE* = 10) for the probe.

**Figure 3.**
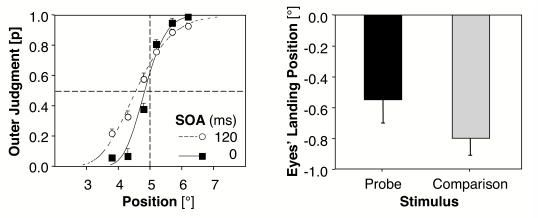
Left: Mean probabilities (and standard errors between participants) for outer judgements of the probe (relative to the 5° mid-position of the comparison stimulus) as a function of the stimulus onset asynchrony (SOA). Curves are fitted functions of a Probit Analysis. A shift to the left indicates PSE (the point of subjective equality) values lower than the objective mid-position and thus a tendency to more outer judgements of the probe. Right: Mean deviations (and standard errors between participants) of eyes’ landing position to the probe and the mid-position of the comparison stimulus. Negative values indicate the amount of saccadic undershoot (Experiment 1, *N* = 22).

The mean PSE values showed that participants tended to judge the probe as being more peripheral than the mid-position of the comparison stimulus. In what follows negative deviations represent PSE values lower than the objective mid-position between comparison stimulus and probe and indicate a tendency towards more outer judgements for the probe. The mean PSE values deviate from the objective mid-position by –0.15°, *SE* = 0.04, *t*(21) = 3.38, *p* < .01, with an SOA of 0 ms and by –0.44°, *SE* = 0.07, *t*(21) = 6.39, *p* < .001, with an SOA of 120 ms. Thus, the tendency to more outer judgements for the probe was present with and without an SOA. The difference between the two PSE values is, however, highly significant, *t*(21) = 4.39, *p* < .001, always two–tailed; cf. Figure 3 (left).

**Figure 4.**
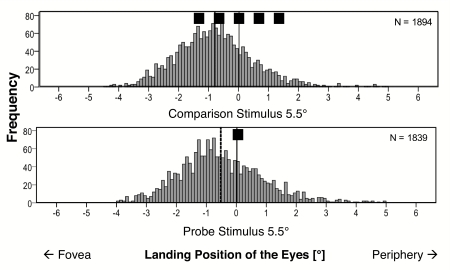
Frequency plots of the horizontal eyes’ landing positions for comparison stimulus (top) and probe (bottom). The dotted lines indicate the means of the histograms (Experiment 1, *N* = 22).

Figure 4 shows the frequency plots of the eyes’ horizontal landing positions. Negative values represent saccadic undershoots in visual angle; positive values represent saccadic overshoots. In general, more undershoots than overshoots were observed for both the comparison and the probe. Additionally, the mean deviations between the eye’s landing position of the first saccade and the real target position revealed a larger undershoot for the comparison stimulus than for the probe. The average undershoot with respect to the real target position is –0.55° for the probe, *SE* = 0.15, *t*(21) = 3.72, *p* = .001; and –0.80° for the comparison stimulus, *SE* = 0.11, *t*(21) = 7.52, *p* < .001. A *t*–test revealed a nearly significant difference between the saccadic undershoot to the mid–position of the spatial extended comparison stimulus and to the less extended probe, *t*(21) = 2.04, *p* = .054 (cf. Figure 3, right part).

### Discussion

 The results of the relative judgement task successfully replicated previous findings (Müsseler et al., 1999; Müsseler & van der Heijden, 2004): The probe is localized as being more peripheral than the midpoint of the comparison stimulus. This tendency is present with an SOA, but also with a simultaneous presentation of both stimuli. Up to now, more outer judgements for the probe were mainly observed with an SOA, but slight tendencies with simultaneous presentation were also observed and reported by Müsseler et al. (1999). In line with the previous research, the outer judgements were clearly more pronounced with an SOA between stimuli than with an SOA of 0 ms. 

The eye-movement data showed that the first saccade undershot both targets. This is in accordance with previous eye-movement studies (e.g., Aitsebaomo & Bedell, 1992; Becker, 1972; Deubel et al., 1984; Henson, 1978). Of special importance in the present context is the (nearly significant) difference between the undershoots to the comparison stimulus and the probe. As expected, a stronger undershoot occurred with saccades to the mid-position of the comparison stimulus than with saccades to the probe (see also Findlay et al., 1993).

A recent model of saccadic programming by Godijn and Theeuwes (2002) can account for the more pronounced undershoot observed with the extended comparison stimulus. It basically suggests that saccades are programmed in a common salience map, in which activity at a specific location spreads to neighbouring locations but inhibits distant locations. The integration of activation might take place in the intermediate layer of the superior colliculus, which receives input from the frontal eye fields, supplementary eye fields, and posterior parietal cortex (cf. Trappenberg, Dorris, Munoz, & Klein, 2001). The preference of the inner squares can be assumed to originate from an increased sensitivity within the saccadic map as a function of eccentricity (Findlay & Walker, 1999). As a consequence, the inner edge of the comparison stimulus receives higher activation to the mean of integrated activation than the outer edge. Accordingly, the eyes could be captured more often by the inner squares.

In the present context it is important to note that the amount of eyes’ undershoot was similar to the foveal mislocalization with the absolute cursor pointing task used by Müsseler et al. (1999, Experi-ment 4, where it was –0.4° for the probe and –0.52° for the comparison stimulus). Moreover, the difference between the mean undershoots to the probe and the comparison stimulus is in the same range of magnitude as the difference between PSE values with and without SOA; (–0.55) – (–0.80) = 0.25° versus (–0.15) – (–0.44) = 0.29°. This could be interpreted as a hint for a correspondence between the perceptual judgement task and the oculomotor task. However, since the difference between probe and comparison stimulus is only marginally significant in the saccadic behaviour, this conclusion needs further evidence from subsequent experiments.

## EXPERIMENT 2

Experiment 1 provided support for the assumption of Müsseler et al. (1999) that the phenomena observed in a relative judgement task are explainable in terms of absolute localization performances. Clearly, this idea needs further supporting evidence. In Experiment 2 we therefore examine whether another well established result obtained with the relative judgement task corresponds with the saccadic eye-movement behaviour: Varying the eccentricity of comparison and probe in the relative judgement task, it appears that the relative mislocalizations increase with increasing eccentricity (see Müsseler et al., 1999, Experiment 3). If the assumption is correct, that the relative mislocalization originates from differences in absolute localization of comparison and probe, one has to assume that an increase in eccentricity does not affect the localization of comparison stimulus and probe equally, that is additively. If the comparison stimulus and the probe are equally affected by eccentricity, the relative mislocalization should remain constant. To explain the increase in mislocalizations with increasing eccentricity it has to be assumed that either the comparison stimu lus is more affected by this manipulation or that the probe is affected less.

For the saccadic eye movement data this entails that only a non-additive pattern of results, indicating that the amount of undershoot increases differentially across eccentricity, would be in correspondence with the relative judgements. The slope of the function relating undershoot to eccentricity has to be steeper with the spatially extended comparison stimulus than with the less extended probe (or to be flatter with the probe, respectively). In other words, a stronger increase in the saccadic undershoot for the comparison stimulus with more eccentric stimulus presentation should be present. Only such a pattern of results could be linked to the observed eccentricity effect with relative judgements. Accordingly, we expected an interaction between eccentricity and target type.

It is worthwhile to note here that the expected non-additive pattern of saccadic eye movements is not the pattern expected given the data from basic eye movement research. From saccadic eye-movement studies it is known that saccades tend to undershoot a target by about 5–10% of its eccentricity (see the Introduction section). When saccades always undershoot the targets by about this amount, the functions relating undershoot to eccentricity should have the same slope for comparison stimulus and probe.

### Method

#### Stimuli, Design, and Procedure

These were the same as in Experiment 1, except for the following changes. In the judgement task all stimuli were presented with an SOA of 120 ms. The mid-position of the comparison stimulus was presented at an eccentricity of either 3.5° or 6.5°. Accordingly, the probe was presented at 2.3°, 2.8°, 3.3°, 3.7°, 4.2°, or 4.7° with a mid-position of the comparison stimulus at 3.5° or was presented at 5.3°, 5.8°, 6.3°, 6.7°, 7.2°, or 7.7° with a mid-position of the comparison stimulus at 6.5°. There were eight repetitions (8 blocks with 24 trials) per participant per cell. In total, the participants received 192 trials.

In the saccade task, the comparison stimulus and the probe were presented in separate blocks. The stimuli could appear either at 3.5° or at 6.5° to the left or to the right of the fixations cross. Sixteen repetitions were gathered for each cell of the design, yielding a total of 128 trials per participant. If no saccade was detected or the latency of the saccade was above 250 ms, an error message appeared. If those errors exceeded 8 trials, one block of 16 trials was added to the experiment. Eye-movement calibration was repeated after two blocks.

The experiment lasted approximately 45 min, including calibrations, training trials, and breaks.

#### Participants

Twenty-six female and 9 male individuals, ranging in age from 16 to 37 years (mean age of 23.14 years), were paid to participate in the experiment.

### Results

Mean relative mislocalization and mean saccadic amplitude were computed separately per participant and eccentricity. Two observers were excluded from the analysis, because their mean values exceeded the criterion of ±2 standard deviations between participants. The mean saccade latency was 172 ms (*SE* = 4) for the comparison stimulus and 171 ms (*SE* = 4) for the probe.

**Figure 5.**
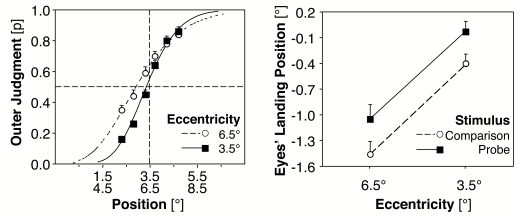
Left: Mean probabilities for outer judgements of the probe as a function of stimulus eccentricity. Right: Mean deviations of eyes' landing position to the probe and the comparison stimulus as a function of eccentricity (Experiment 2, *N* = 33).

In the judgement task PSE values indicated a more pronounced tendency to outer judgements at the eccentricity of 6.5° than at the eccentricity of 3.5°, *t*(32) = 5.01, *p* < .001 (cf. Figure 5, left part). At 6.5° the PSE value indicates a significant difference from the objective mid-position, –0.59°, *SE* = 0.13, *t*(32) = 4.51, *p* < .001. At 3.5° this result was only marginally significant, –0.12°, *SE* = 0.08, *t*(32) = 1.49, *p* = .15.

**Figure 6.**
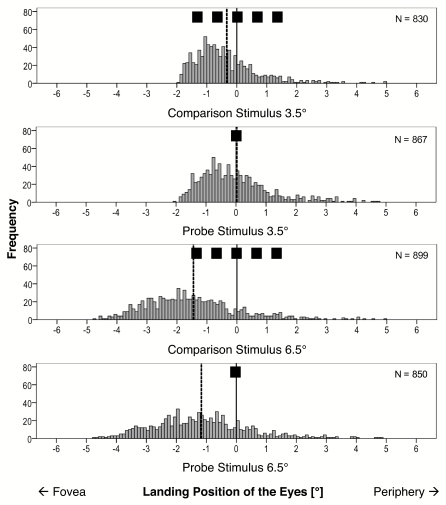
Frequency plots of the horizontal eyes' landing positions for comparison stimulus and probe at 3.5º and 6.5º eccentricity. The dotted lines indicate the means of the histograms (Experiment 2, single-target presentation, *N* = 33).

Figure 6 shows the frequency plots of the eyes’ horizontal landing positions. For the saccade task the deviations in saccadic amplitude from the objective positions were entered in a 2 (comparison stimulus vs. probe) x 2 (3.5° vs. 6.5° eccentricity) analysis of variance (ANOVA). The analysis revealed a significant effect of type of stimulus, comparison stimulus, and probe, *F*(1, 32) = 6.1, *MSE* = 0.83 , *p* < .05; the saccadic undershoot to the comparison stimulus is more pronounced than the undershoot to the probe (cf. Figure 5, right part). Further, the amount of undershoot increases with eccentricity, *F*(1, 32) = 223.6, *MSE* = 0.16, *p* < .001. The interaction between the factors type of stimulus and eccentricity was far from significant (*p* > .20).

### Discussion

 In the judgement task, the results again replicated the basic finding of Müsseler et al. (1999) that the probe is localized as being more peripheral than the mid-point of the comparison stimulus. Moreover, and of more importance in the present context, the results replicated the finding obtained by Müsseler et al. (Experiment 3) that showed that the relative mislocalization increases with increasing eccentricity. 

In the saccade task undershoots were observed with the probe and with the comparison stimulus. Moreover, the amount of undershoot was significantly larger with the comparison stimulus than with the probe. This finding replicates and thereby substantiates the marginally significant result obtained in Experiment 1.

The size of the saccadic undershoot increased with increasing eccentricity. The interaction between type of stimulus and eccentricity was, however, not significant; an additive effect of eccentricity for comparison stimulus and probe was found. This additivity is in line with the results reported by basic eye movement research: The undershoot is a fixed percentage of target eccentricity (see e.g., Deubel, 1999; see also the Introduction section). Of course, this outcome does not come as a surprise. In the saccadic eye movement task, exposure conditions were used that were virtually identical to those used in basic single-target saccadic eye movement research (see e.g., Deubel, 1999).

Note, however, that the additivity of the factors stimulus type and eccentricity is not in accordance with the assumption that absolute position judgements are at the basis of the phenomena observed in the relative judgement task. In the relative judgement task an eccentricity effect is observed: Relative mislocalization increases with increasing eccentricity. This eccentricity effect is not apparent in the saccadic eye movement behaviour: Contrary to our predictions the difference between undershoots to comparison stimulus and probe remains the same with increasing eccentricity. Possibly the absence of the interaction indicated a dissociation between saccadic behaviour and relative judgement, but it may be worthwhile to re-analyse our conditions.

So far, our considerations were based on the assumption that in the relative judgement task the probe and the comparison stimulus independently determine the direction and size of a saccadic eye movement. That is why in the saccadic eye movement task we used the single-item exposure conditions used in basic eye movement research. However, it cannot be excluded that in the relative judgement task, where a probe and a comparison stimulus are presented in close temporal proximity, the spatial codes of comparison stimulus and the probe modulate each other. If that is true, the additional presentation of the context stimulus could also affect the saccadic behaviour. This is tested in the subsequent experiment.

## EXPERIMENT 3

The results obtained in the saccadic eye-movement task in Experiment 2 are in accord with those reported by basic saccadic eye movement research: No interaction is found between stimulus type and eccentricity. The results are, however, not compatible with Müsseler et al.’s explanation (1999) of the phenomena observed in the relative judgement task. For the eccentricity effect observed in the relative judgement task that explanation requires an interaction between stimulus type and eccentricity in the eye-movement task.

In the saccadic eye-movement task of Experiment 1 (and 2), single stimuli, either the probe or the comparison, were used as targets. In the relative judgement task, however, the two stimuli were presented in close temporal contiguity. The probe is presented in the context of the comparison stimulus and context effects are well known in saccadic eye-movement research. For example, saccades tend to land at an intermediate position between a target and a distractor (Findlay, 1982). It can therefore not be excluded that the context modulates the saccadic eye movements to comparison stimulus and probe.

Experiment 3 was conducted to examine this possibility. Like in the judgement task, both stimuli were now presented in each trial of the saccade task with the saccadic target determined blockwise as either the comparison stimulus or the probe. If the saccades show the predicted non-additive pattern of undershoots, there is again a correspondence between saccadic behaviour and perceptual relative judgements.

Additionally, the number of squares of the comparison stimulus were increased from five to seven to stress the different spatial extension of the stimuli. The relative mislocalization was shown to increase with the spatial extension of the comparison stimulus (Müsseler et al., 1999, Experiment 5). Measuring the saccadic amplitudes under these conditions offers the possibility to test our assumptions over a wider spatial range.

### Method

#### Stimuli, Design, and Procedure

The stimuli, design, and procedure were the same as in Experi-ment 1 except for the following changes. In both tasks, the comparison stimulus now consisted of seven squares instead of five squares, that is, the extension changed from 3° to 4.3°. The most important change was introduced in the saccade task: As in the judgement task in both conditions – saccade to the probe and saccade to the comparison – both the comparison stimulus and the probe were presented separated by an SOA of 120 ms.

In the saccade task, two different instructions were given in two blocks of trials with the order of instruction counterbalanced over participants. In one block the participants were asked to make a saccade to the mid-position of the comparison stimulus, and in the other block to make a saccade to the probe and to ignore the other stimulus.

The midpoint of the comparison stimulus was at an eccentricity of either 3.5° or 6.5° (the position of the probe was varied as in Experiments 1 and 2 with steps of ± 0.5°). In total, the participants received 320 trials in both tasks. The experiment lasted approximately 45 min.

#### Participants

Twenty-one female and 9 male individuals who ranged in age from 20 to 39 years (mean age of 25 years) were paid to participate in the experiment.

### Results

Mean relative mislocalizations and mean saccadic amplitudes were computed per participant and condition. Two participants were excluded because their mean PSE values or saccadic amplitudes deviated more than ±2 standard deviations from the other participants. The mean saccade latency was 248 ms (*SE* = 7) for the comparison stimulus and 122 ms (*SE* = 7) for the probe. This obvious latency difference might originate from the tendency to initiate the saccade to the comparison stimulus not before both stimuli were presented and/or from the tendency to use the comparison stimulus as a temporal cue to initiate the saccade to the target.

**Figure 7.**
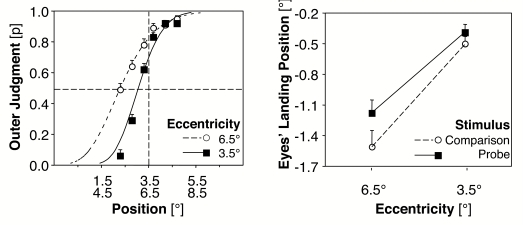
Left: Mean probabilities for outer judgements of the probe as a function of stimulus eccentricity. Right: Mean deviations of eyes' landing position to the probe and the comparison stimulus as a function of eccentricity (Experiment 3, *M* = 28).

In the judgement task a *t*-test revealed a significant difference between PSE values for the two eccentricities, *t*(27) = 10.82, *p* < .001 (cf. Figure 7, left part). At 3.5° the deviation from the objective mid-position was –0.44°, *SE* = 0.08, *t*(27) = 5.46, *p* < .001; and at 6.5° the deviation was –1.09°, *SE* = 0.10, *t*(27) = 10.69, *p* < .001.

**Figure 8.**
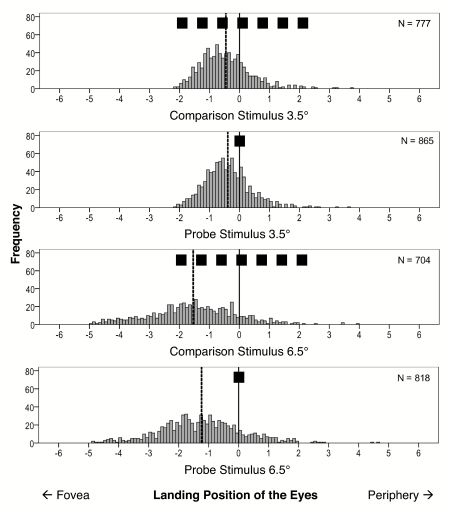
Frequency plots of the horizontal eyes' landing positions for comparison stimulus and probe at 3.5° and 6.5° eccentricity. The dotted lines indicate the means of the histograms (Experiment 3, successive presentation of both stimuli, *N* = 28).

Figure 8 shows the frequency plots of the eyes’ horizontal landing positions. The mean deviations of the saccadic amplitudes from the objective target positions were entered as dependent variable in a 2 (comparison stimulus and probe) x 2 (eccentricity of 3.5° and 6.5°) ANOVA. The analysis revealed significant effects of type of target, *F*(1, 27) = 7.3, *MSE* = 0.19 , *p* = .01; eccentricity, *F*(1, 32) = 78.0, *MSE* = 0.29, *p* < .001; and interaction between type of target and eccentricity, *F*(1, 27) = 6.8, *MSE* = 0.05, *p* = .02 (cf. Figure 7, right part). The saccadic undershoot to the comparison stimulus is more pronounced than the undershoot to the probe; the undershoot increases with eccentricity, and this increase is more pronounced for the comparison stimulus than for the probe.

### Discussion

In the judgement task the probe was again localized as being more peripheral than the comparison stimulus and the amount of mislocalization increased when the eccentricity of presentation was increased. These results replicate the finding reported by Müsseler et al. (1999, Experiment 3). Moreover, with the present comparison stimulus of seven squares the amount of mislocalization was clearly larger than in Experiment 2, where the comparison stimulus consisted of five squares. The mean PSE values were –0.355° (Experiment 2) and –0.765° (Experiment 3), respectively, *SE* = 0.132, *t*(59) = 3.15, *p* = .003. This outcome replicates the result reported by Müsseler et al. (1999, Experiment 5).

The saccade task revealed the most important finding. With the additional presentation of the context stimulus, the saccadic undershoots showed the predicted non–additive interaction. The difference between the undershoots for comparison stimulus and probe was larger at 6.5° than at 3.5° eccentricity. In contrast, in Experiment 2 with a single-target presentation no comparable difference occurred. Apparently, the presentation of the task-irrelevant context stimulus leads to a pattern of saccadic undershoots that matches with the observed eccentricity effect in the perceptual judgement task. The context stimuli appear to modulate the saccadic eye movements to the targets, thus producing the pattern of results required for the explanation (given by Müsseler et al., 1999) of the eccentricity effect observed in the relative judgement task.

## GENERAL DISCUSSION

Müsseler et al. (1999) investigated spatial localization with a relative judgement task. The observers were asked to judge the peripheral position of a small probe with respect to the mid-position of a spatially extended comparison stimulus. When the two stimuli were flashed successively, the observers perceived the small probe as being more peripheral than the mid-position of the comparison stimulus. In the present study this outcome, plus a number of additional related phenomena reported by Müsseler et al. (such as *the extension effect* and *the eccentricity effect*), was replicated.

To explain the relative mislocalization, the authors assumed that it emerged from different absolute localizations of probe and comparison stimulus; the exact assumption was that both the probe and the comparison stimulus are perceived more foveally than they really are and that the spatially extended comparison stimulus is even perceived more foveally than the spatially less-extended probe.

Saccadic eye movements to a target position can be regarded as absolute judgement of the target location. A pattern of results as specified in the explanatory assumption proposed by Müsseler et al. (1999) has been reported by basic saccadic eye movement research: Saccadic eye movements tend to undershoot the target (e.g., Aitsebaomo & Bedell, 1992; Bischof & Kramer, 1968; Lemij & Collewijn, 1989), and the undershoot seems to be greater with spatially extended stimuli than with less extended stimuli (e.g., Findlay et al., 1993). Saccadic eye movements have, however, up to now never been investigated in the experimental setting used in the relative judgement task. Therefore the aim of the present study was to examine in one experimental setup whether the target positions as indicated by the saccadic eye movements correspond with the absolute positions presupposed by the discussed explanation (Müsseler et al., 1999) of the phenomena observed in the relative judgement task.

The basic results obtained in the saccadic eye-movement tasks support the main idea of Müsseler et al.: In all three experiments reported here, the saccadic eye movements undershoot both the comparison stimulus and the probe. Moreover, they undershoot the comparison stimulus even more than the probe. Also the extension effect was clearly apparent in the saccadic eye movement data (see the comparison between Experiment 2 and 3 in the Discussion of Experiment 3). A problem was, however, encountered with the eccentricity effect. This problem requires some further discussion.

The pattern of saccadic eye movements required for explaining the eccentricity effect only showed up in Experiment 3 where both comparison and probe were presented in close temporal proximity; in this experiment an interaction between type of target (probe and comparison) and eccentricity (3.5º and 6.5º) was found. This interaction was absent in Experiment 2 with isolated blockwise presentation of comparison stimulus and probe. When comparing these experiments, it is obvious that the critical difference between them is target selection. In the saccadic eye movement task of Experiment 2, on each trial after the disappearance of the fixation point, a single target (the comparison stimulus or the probe) appeared in an otherwise empty field. In this exposure situation target selection is no problem at all. The situation mimics the single-stimulus situation used in basic saccadic eye movement research. That research consistently reports a 5–10% undershoot. With such a fixed undershoot an additive relation between type of target and eccentricity is to be expected, independently of how the difference between types of targets is produced.

In the saccadic eye movement task of Experiment 3, in each trial after the disappearance of the fixation point, two stimuli, the comparison stimulus and the probe, appeared in close temporal proximity. In the instruction before a block of trials it was verbally specified whether the comparison stimulus or the probe should be regarded as the target for the eye. In other words, this task requires the participant to make a top-down selection of the target and to ignore a distractor. However, it is well known that distractors affect pointing tasks and eye-movement tasks (e.g., Sheliga, Riggio, Craighero, & Rizzolatti, 1995; Tipper, Howard, & Jackson, 1997). It is likely, because of the decreasing retinal acuity, that these tendencies increase with increasing eccentricity. Therefore, in this situation an interaction between type of target and eccentricity can arise.

In the present context it is of importance to see that the information processing situation in the relative judgement task is closer to the experimental situation in the saccadic eye movement task of Experiment 3 than that of Experiment 2. Just as in the saccadic eye movement task of Experiment 3, in the relevant conditions of the relative judgement tasks in each trial, both comparison stimulus and probe are presented in close temporal proximity. Moreover, just because the positions of the comparison stimulus and the probe have to be compared, top-down selection is required.

Taken all together, the main outcome of the saccadic eye-movement research here reported is clearly in accord with, and therefore supports, the explanatory assumption introduced by Müsseler et al. (1999) for accounting for the main phenomena observed in the relative judgement task (see above). Also the eccentricity effect can be accounted for because the eye movement data of Experiment 3, not those of Experiment 2, are the relevant data.

As already stated in the Introduction, the fact – now further supported by the data presented here – that saccadic eye movement research supports the assumptions made by Müsseler et al. suggests an intriguing possibility: The possibility that the saccadic eye movement system is at the basis of, and provides the information for, position judgements in position judgement tasks (see also, e.g., van der Heijden, Müsseler, & Bridgeman, 1999; Wolff, 1987, for this suggestion). If that is correct, the difference between the absolute localizations of the stimuli should correspond not only qualitatively but also quantitatively with the relative localizations. This is examined in the subsequent analysis.

In the present study the landing positions of the eyes to the comparison stimulus and the probe, which are used as indicators of the perceived absolute localizations, proved to be determined by various variables (above all by the eccentricity, the spatial extension, and the context). Correspondingly, the differences of the landing positions of the eyes determined by these variables should correspond with the PSE values from the relative judgement task, which also proved to be determined by these variables.

In order to compare the correspondence more directly and to ensure the generalization of the data, the subsequent analysis is based on two steps:

(1) Multiple Linear Regression is used to estimate the saccadic landing positions determined by the various variables.

(2) Then the differences of the estimated landing positions are compared with the PSE values of the present and previous experiments.

### Multiple Regression analysis

Previous research revealed that saccadic amplitudes are determined by several variables. In the present context the most relevant variables are the eccentricity of stimulus presentation (see also Aitsebaomo & Bedell, 1992; Bischof & Kramer, 1968; Lemij & Collewijn, 1989), the spatial extension of the stimuli (see also Findlay et al., 1993), and the context of stimuli (see also Findlay, 1982). The variables proved also to determine saccadic amplitudes in the present Experiments 1–3.

To estimate the contribution of each variable to the saccadic amplitude, these variables are entered as predictor variables in a Multiple Linear Regression (MLR). Multiple Regression provides information on how the saccadic amplitude (the criterion variable) is determined quantitatively by the predictor variables. The measure for the relative impact of the predictors on the criterion is the respective slope ß. In its non-standardized form, ß reports the increase (or decrease) in saccadic amplitude in units of the predictor variables.

The following values of predictor variables are entered in the MLR: the eccentricity of stimulus presentation with the values of 3.5 or 6.5°, and the spatial extension of the stimuli with the values 0.165° for the probe and 1.5° (Experiment 2) or 2.11° (Experiment 3) for the comparison stimulus,^2^ while the context describes the presence or absence of the second stimulus. In Experiment 2 no context stimuli were presented (context = 0), in contrast to Experiment 3, where the second stimulus serves as the context for the other stimulus (context = 1). Additionally, Experiment 3 revealed an interaction between eccentricity and extension. This interaction can be taken into account by calculating the product of the two predictor variables and entering this into the regression analysis as an additional variable (e.g., Kerlinger & Pedhazur, 1973, p. 415).

The mean saccadic amplitudes of the conditions of Experiments 2 and 3 were entered as the criterion variable in a Multiple Linear Regression.^3^ The analysis yields a multiple *R*² of .994 and the equation:

Saccadic amplitude = 0.699 x Eccentricity – 0.056 x Stimulus extension – 0.108 x Context – 0.023 x (Eccentricity x Extension) + 0.911

In other words, this equation allows us to estimate with high precision the saccadic landing positions. As expected, eccentricity contributes to saccadic amplitude to a large degree and the contribution of stimulus extension, context, and the interaction only modify the widths of the amplitudes. Nevertheless, based on this equation, we can estimate the amplitudes to the probe and the comparison stimulus in all our experiments and we were able to compare them directly with the perceptual judgements.

### Comparison of estimated and observed relative mislocalizations for the present and previous experiments

The observed relative mislocalization was assumed to originate from the different absolute localizations of comparison stimulus and probe. Thus, the difference in saccadic amplitudes to the comparison stimulus and the probe can be used as an estimation of the observed relative mislocalization.

**Figure 9.**
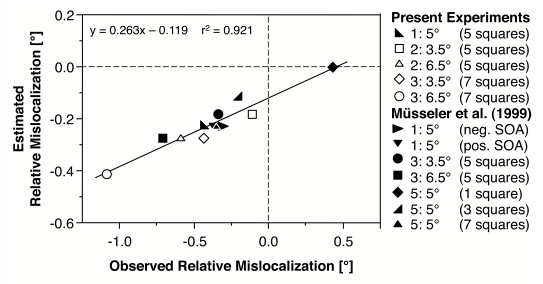
Regression between observed and estimated relative mislocalization. Estimated relative mislocalizations are based on the difference in saccadic amplitudes to the comparison stimulus and the probe. Light symbols represent the experiments on which the Linear Multiple Regression is based (Experiments 2 and 3). Dark symbols represent Experiment 1 and other experiments with relative judgements by Müsseler et al. (1999).

Figure 9 shows the plot of the observed and the estimated mislocalizations of the present experiments as well as of three further experiments, which were gathered under comparable conditions (Müsseler et al., 1999, Experiments 1, 3, and 5). Linear regression revealed an *R*² of .921. This result demonstrates that the mislocalization estimated from the saccadic behaviour fits nicely with the mislocalization observed in the relative judgement task. The linear function integrates all effects of the different eccentricities and of the different spatial extensions of comparison stimuli.

However, the slope of the regression line is not 1 and the intercept is not 0. Especially the deviation of the slope indicates that the observed mislocalization is more pronounced than the estimated mislocalization derived from the landing positions of the eye movements. According to the proposed distinction between vision for perception and vision for action (Milner & Goodale, 1995), this is what to expect. Recent studies testing this distinction revealed only small effects of an illusion on action scaling as compared to its effect on perception (e.g., Bartelt & Darling, 2002; Haffenden, Schiff, & Goodale, 2001). Another explanation of the rather small slope is that it emerges from a range effect in saccades. Within our experiments, stimuli were always presented at a constant range of eccentricity. This might have led to comparatively large saccadic amplitudes with small eccentricities and small saccadic amplitudes with large eccentricities. Such a range effect in saccades is already known from the literature (e.g., Kapoula, 1985) and it is possible that it artificially reduced the differences between saccadic amplitudes. Future research is clearly needed to clarify this detail of our results.

In sum, the present findings provide evidence for the account that the relative mislocalization is based on differences in absolute localizations, which might originate from the eye-movement system. We have already speculated that the system in charge of the guidance of saccadic eye movements is also the system that provides the metric in perceived visual space (Müsseler & van der Heijden, 2004; van der Heijden, Müsseler, & Bridgeman, 1999; see also e.g., Bruno & Morrone, 2007; Collins et al., 2007; Georg & Lappe, 2009; Koenderink, 1990; Wolff, 1987). According to this view the system of sensation and eye movement organizes itself *via* an interaction with the environment, which, after all, establishes spatial perception.

## FOOTNOTES

1 Additionally, an as yet unpublished experiment with and without eye-movement instruction did not indicate an effect of eye movements on the relative mislocalization observed between comparison stimulus and probe.

2 The participants’ task was to gear their eyes to the mid-position of the stimuli, that is 0.33/2 = 0.165° for the probe and 3/2 = 1.5° (Experiment 2) or 4.22/2 = 2.11° (Experiment 3) for the comparison.

3 As previous research and the present experiments had shown that all variables contribute significantly to saccadic amplitude, we abstained from reporting significances of the regression analysis. Instead, what counts in the present context is the explained variance of the regression.
